# Impact of intranasal cigarette smoke exposure on murine peripheral immune responses and lung pathology

**DOI:** 10.3389/ftox.2026.1884554

**Published:** 2026-08-03

**Authors:** Laura G. Bartlett, Yasmin Azizbayli, Amanda L. Tatler, Adam J. Watkins, Lucy C. Fairclough

**Affiliations:** 1 School of Life Sciences, The University of Nottingham, Nottingham, United Kingdom; 2 School of Medicine, The University of Nottingham, Nottingham, United Kingdom; 3 Division of Clinical Medicine, School of Medicine and Population Health, University of Sheffield, Sheffield, United Kingdom

**Keywords:** chronic obstrucive pulmonary disease, cigarette smoke, immunopathology, intranasal dosing, mouse model

## Abstract

Chronic obstructive pulmonary disease (COPD) is driven primarily by cigarette smoke exposure, yet modelling its immunopathogenesis in mice is complicated by systemic stress and welfare concerns associated with conventional exposure models. Here, we employ a refined intranasal cigarette smoke extract (CSE) model to examine pulmonary pathology and peripheral immune responses in male and female C57BL/6J mice. Repeated intranasal CSE exposure over 12 weeks was well tolerated and induced dose-dependent pulmonary neutrophilia, airway remodelling, and collagen deposition, with more pronounced pathology observed in female mice at higher exposure levels. In contrast, longitudinal multiparametric flow cytometric analysis revealed no consistent dose-dependent alterations in peripheral immune cell composition or activation, as confirmed by both conventional gating and unbiased clustering approaches. These findings demonstrate that COPD-like lung pathology can arise in the absence of overt systemic immune disruption and highlight the importance of lung inflammatory processes in disease development. This refined intranasal model provides a welfare-conscious platform for studying pulmonary immunopathology in COPD.

## Introduction

Chronic obstructive pulmonary disease (COPD) is a progressive respiratory disorder characterised by persistent airflow limitation, chronic inflammation, and irreversible structural remodelling of the airways and lung parenchyma (Corlateanu et al., 2020). While a range of noxious particulate exposures have been associated with COPD, prolonged exposure to cigarette smoke is considered the dominant aetiological factor (Adeloye et al., 2022; Mannino et al., 2006). Despite its significant global burden, therapeutic options for COPD remain limited, largely focusing on symptom management and mitigation of acute exacerbations rather than disease modification, which reflects the incomplete understanding of the pathogenesis of COPD.

Mechanistically, the pathophysiology and persistence of COPD are associated with structural alterations to the pulmonary environment. This can occur through alterations to the extracellular matrix (ECM) and chronic inflammation, which ultimately lead to significant airway remodelling. Proportional reductions in key ECM and ECM-associated proteins within the parenchyma and airway walls, including lumican, collagen I, fibulin-2, decorin, versican, and LTBP4, have been shown to correlate with decreased FEV_1_. These disruptions in ECM signatures are associated with disease presence and severity, supporting the concept that coordinated disruption of ECM networks, rather than isolated protein loss, contributes to structural and functional deterioration in COPD lungs (Joglekar et al., 2024). Such changes can contribute towards hypoxic environments and endothelial damage of vascular and airway components, leading to increased pulmonary inflammation (Lodge et al., 2022). Damage to the endothelial lining of airways leads to altered epithelial layer composition, mucus hypersecretion and poor mucosal clearance, which are associated with immune cell accumulation that contributes to a persistent inflammatory microenvironment (Hogg et al., 2004; Koo et al., 2018).

In both human disease and experimental models, chronic immune cell accumulation within the lungs is accompanied by qualitative alterations in immune function, including aberrant cytokine signalling and impaired resolution of inflammation. Pulmonary neutrophilia is a hallmark of COPD and contributes directly to tissue damage and remodelling through protease release and heightened pattern recognition receptor signalling, which further amplifies neutrophil recruitment and activation within the lung microenvironment (Pouwels et al., 2017; Sin and Van Eeden, 2012). Accumulated macrophages similarly promote persistent inflammation through altered effector function and sustained production of proinflammatory mediators that facilitate immune cell trafficking and extracellular matrix degradation (Boorsma et al., 2013; Lee et al., 2021). Dysregulated innate immune activity, in turn, promotes pathogenic adaptive immune responses, with increased pulmonary CD4^+^ T-cell accumulation associated with disease severity and driven in part by an imbalance between pro-inflammatory and regulatory T-cell subsets (Kheradmand et al., 2023; Paats et al., 2012; Qin et al., 2022). Together, these maladaptive innate and adaptive immune processes reinforce a self-perpetuating inflammatory microenvironment that underpins disease persistence and progression in COPD. Mechanistic dissection of these immunopathogenic pathways therefore relies on experimental models that preserve immune fidelity and enable interrogation of immune–tissue interactions without confounding systemic effects.

Experimental mouse models have been central to advancing mechanistic understanding of COPD pathogenesis, particularly for interrogating immune-mediated processes that cannot be readily examined in human patients. Commonly employed approaches include chronic cigarette smoke exposure and elastase-induced emphysema models. Elastase-based models efficiently induce alveolar destruction but rely on acute chemical injury, bypassing immune-driven disease development and limiting their relevance for studying chronic immunopathogenic mechanisms (Rydell-Törmänen et al., 2019). Cigarette smoke exposure models more closely reflect the primary aetiological driver of COPD, however, they frequently induce substantial physiological stress, weight loss, and systemic illness, which complicate interpretation of immune responses (Chow et al., 2018; Serre et al., 2021). Because immune cell distribution, activation state, and cytokine signalling are highly sensitive to systemic stress, these confounding effects present a significant challenge when analysing immune mechanisms, particularly peripheral immune responses. As a result, there remains a need for refined cigarette smoke exposure models that preserve immune fidelity while enabling detailed assessment of both pulmonary pathology and immune alterations.

In this study, we investigate the impact of intranasal cigarette smoke exposure on murine lung pathology and immune responses using a refined experimental approach designed to minimise systemic stress. We aim to characterise the structural and inflammatory consequences of cigarette smoke exposure within the lung, alongside associated alterations in peripheral immune cell populations. By integrating histological assessment of lung pathology with multiparametric flow cytometric analysis of immune responses, this work seeks to establish the utility of intranasal cigarette smoke exposure as a model for studying immunopathogenic mechanisms in COPD while preserving animal welfare and immune interpretability.

## Methods

### Animals and ethical approval

All animal procedures were conducted in accordance with the UK Animals (Scientific Procedures) Act 1986 and approved under a UK Home Office Project Licence (PPL PP5215372). Procedures were performed by appropriately licensed personnel under a Personal Licence, and all work was approved by the University of Nottingham Animal Welfare and Ethical Review Body. Animal care and experimental design adhered to NC3Rs guidelines for refinement and welfare.

Male and female C57BL/6J mice were obtained from Charles River Laboratories and housed in the University of Nottingham BioSupport Unit. Mice were housed in sex-matched groups under controlled temperature and humidity with a 12 h light–dark cycle and *ad libitum* access to food and water. Animals were 8 weeks old on arrival and did not undergo regulated procedures until 11 weeks of age.

Throughout the 12-week exposure period, animal welfare was monitored closely by trained investigators. Mice underwent regular behavioural and physical health assessments, including body weight measurement, body condition scoring, and observation for signs of respiratory distress or altered behaviour. At the end of the study period, mice were culled via intraperitoneal overdose of sodium pentobarbital (Dolethal, 200 mg/mL) at a dose consistent with standard institutional practice (200 mg/kg). Death was confirmed via exsanguination by cutting the femoral artery. No adverse effects requiring early humane endpoints were observed, and all interventions were performed in accordance with approved welfare protocols.

### Intranasal cigarette smoke extract (CSE) generation and exposure

Cigarette smoke extract (CSE) was generated fresh on dosing days using phenol red–free RPMI-1640 medium. Briefly, smoke from a single Marlboro Red cigarette was drawn through 10 mL culture medium to generate a stock solution, which was sterile filtered using a 0.45 μm filter to remove particulate matter. This stock was defined as 10% CSE and diluted in phenol red–free RPMI to prepare working concentrations of 1% and 3% CSE, to reflect a light smoker (∼5 cigarettes per day) and a heavy smoker (∼15 cigarettes per day). The calculations of the cigarette smoke extract used to reflect light and heavy smokers are shown in Supplementary Material S1. Control animals received phenol red–free RPMI-1640 medium alone.

Mice were exposed to CSE by intranasal administration three times per week for 12 weeks. Prior to dosing, animals were briefly anaesthetised with 3% isoflurane and 25 μL of CSE or control medium was delivered intranasally. Mice were monitored during recovery and returned to their home cages following full recovery from anaesthesia.

### Animal welfare and refinement

Mice underwent regular behavioural and physical health assessments prior to intranasal dosing, including evaluation of activity, social interactions, coat condition, body condition score, respiratory pattern, and evidence of injury. Body weight was recorded three times per week and used as a primary indicator of systemic health. To minimise stress associated with handling and procedures, mice were acclimatised to restraint using positive reinforcement prior to study initiation and throughout the exposure period.

### Peripheral blood collection and flow cytometry sample preparation

Peripheral blood was collected longitudinally by tail vein microsampling once per week, with no more than 50 μL taken at one time in accordance with NC3Rs and LASA guidelines. Whole blood was collected into heparinised tubes and processed immediately for flow cytometric analysis.

For immune phenotyping, red blood cells were lysed prior to staining, and then cells were stained using a 22-colour antibody panel (Supplementary Tables S1, S2). For extracellular staining, cells were blocked using Fc receptor blockage, then resuspended in 100 μL PBA containing fluorochrome-conjugated antibodies and were incubated at 4 °C for 30 min in the dark. Cells were washed twice with PBA (300 g, 5 min) and fixed in 500 μL fixation buffer (BD Biosciences, Franklin Lakes, NJ, USA) for 30 min at 4 °C. For intracellular staining, fixed cells were washed with 2 mL 1× Perm/wash buffer (BD Biosciences, Franklin Lakes, NJ, USA; 500 g, 5 min) and were resuspended in 100 μL of Perm/wash buffer containing antibodies against intracellular targets. Samples were incubated at room temperature for 30 min in the dark, washed twice with PBA (500 g, 5 min), and resuspended in 300 μL of fresh fixation buffer for storage at 4 °C until acquisition by flow cytometry. Unstained and single-colour controls were included as appropriate for gating and compensation.

### Flow cytometry and gating strategy

Peripheral immune cell populations were analysed by multiparametric flow cytometry. Data was acquired on a Sony ID7000 flow cytometer at the University of Nottingham Flow Cytometry Facility. A viability dye was used to exclude dead cells prior to analysis. Immune populations were identified through sequential gating of singlets and viable CD45^+^ cells, followed by lineage- and phenotype-specific marker expression to define lymphoid and myeloid subsets.

T cell populations were gated on CD3^+^ populations, which were further differentiated into CD4^+^ and CD8^+^ populations. Each T cell subset was stained for activation and differentiation markers by gating for CCR7, CD25 and CD69. Activation and differentiation were determined by the combination of markers present, with CCR7^+^ or CD69^+^CD25^-^ populations defined as in early activation, CD69^+^CD25^+^ populations as mid activation, and CD69^=^CD25^+^ populations as late activation. CD4^+^CD69^=^CD25^+^ populations were classified as both Tregs in addition to conventionally activated CD4^+^ T cells. Activation was also further defined by the presence of positive staining for IFN-γ, IL-6, IL-17A and TNF-α.

All other cell types were identified from CD3^-^ populations. NK cells were identified from CD3^-^CD49b^+^ populations. Macrophages and neutrophils were defined by gating for CD11b and Ly6G/Ly6C (Gr-1), with CD11b^+^Gr-1^med^ populations defined as monocytes, and CD11b^+^Gr-1^hi^ populations defined as neutrophils. Macrophages were first gated for CD11b and I-A/I-E (MHCII), with double positive populations being further gated for CD80 and CD86. CD80^+^CD86^+^ populations were then gated for CD163, with positive populations defined as macrophages. Dendritic cells and B cells were first gated for CD11b and MHCII, with MHCII^+^CD11b^-^ populations being gated for CCR7 and CD38. CCR7^+^CD38^+^ populations were defined as dendritic cells, and CCR7^-^CD38^+^ populations were defined as B cells.

Data were analysed using FlowJo software, with representative gating strategies shown in Supplementary Figure S1.

### Unbiased clustering analysis

Unbiased clustering of peripheral immune cells was performed using FlowSOM analysis. Viable CD45^+^ single cells were exported from FlowJo and equally sampled across sex and exposure groups prior to clustering. All markers included in the flow cytometry panel were used for analysis. Dimensionality reduction and visualisation were performed using t-distributed stochastic neighbour embedding (tSNE). The ‘Downsample’ plugin was applied to standardise each sample to 10,000 events, minimising batch effects. Samples were concatenated by experimental condition (e.g., sex and exposure group) to generate combined files. The optimal cluster number was estimated using the ‘Phenograph’ plugin. This value was input into the FlowSOM plugin to delineate self-organizing map clusters. Resultant clusters were visualised via tSNE. ‘Cluster Explorer’ was employed to generate annotated t-SNE plots (colour-coded by marker expression or population frequency) and quantify event distributions per cluster. Differences in cluster distribution across experimental variables were assessed by two-way ANOVA.

### Lung tissue processing and histological staining

Following euthanasia, lungs were fixed overnight in 10% neutral buffered formalin, transferred to 70% ethanol, and paraffin embedded using standard protocols. Sections were cut at 10 μm thickness and mounted onto glass slides. Lung morphology and airway structure were assessed using haematoxylin and eosin (H&E) staining. Collagen deposition was evaluated on consecutive sections using Picrosirius red staining.

### Histological imaging and quantitative analysis

Stained sections were imaged at ×20 magnification using a Zeiss AxioScan 7 slide scanner. For analysis, 500 × 500 μm regions of interest were randomly selected from the left lung lobe. Pulmonary neutrophil infiltration was quantified by manual counting within regions containing alveoli, airways, and blood vessels. Alveolar enlargement was assessed by mean linear intercept (MLI) analysis using ImageJ using a semi-automated plugin (Crowley et al., 2019). Five randomly selected alveolar regions per lung were analysed, and average alveolar diameter was calculated for each animal. Quantification of fibrosis in picrosirius red stained sections was performed using a modified Ashcroft score by two independent researchers (Hübner et al., 2008). A mean was taken from the two scores for each mouse/treatment.

### Statistical analysis

Statistical analyses were performed using GraphPad Prism. Data were analysed using two-way analysis of variance (ANOVA) to assess the effects of cigarette smoke exposure and sex, with *post hoc* multiple comparisons performed where appropriate. Data are presented as mean ± standard error of the mean (SEM), and statistical significance was defined as *P* < 0.05.

**FIGURE 1:**
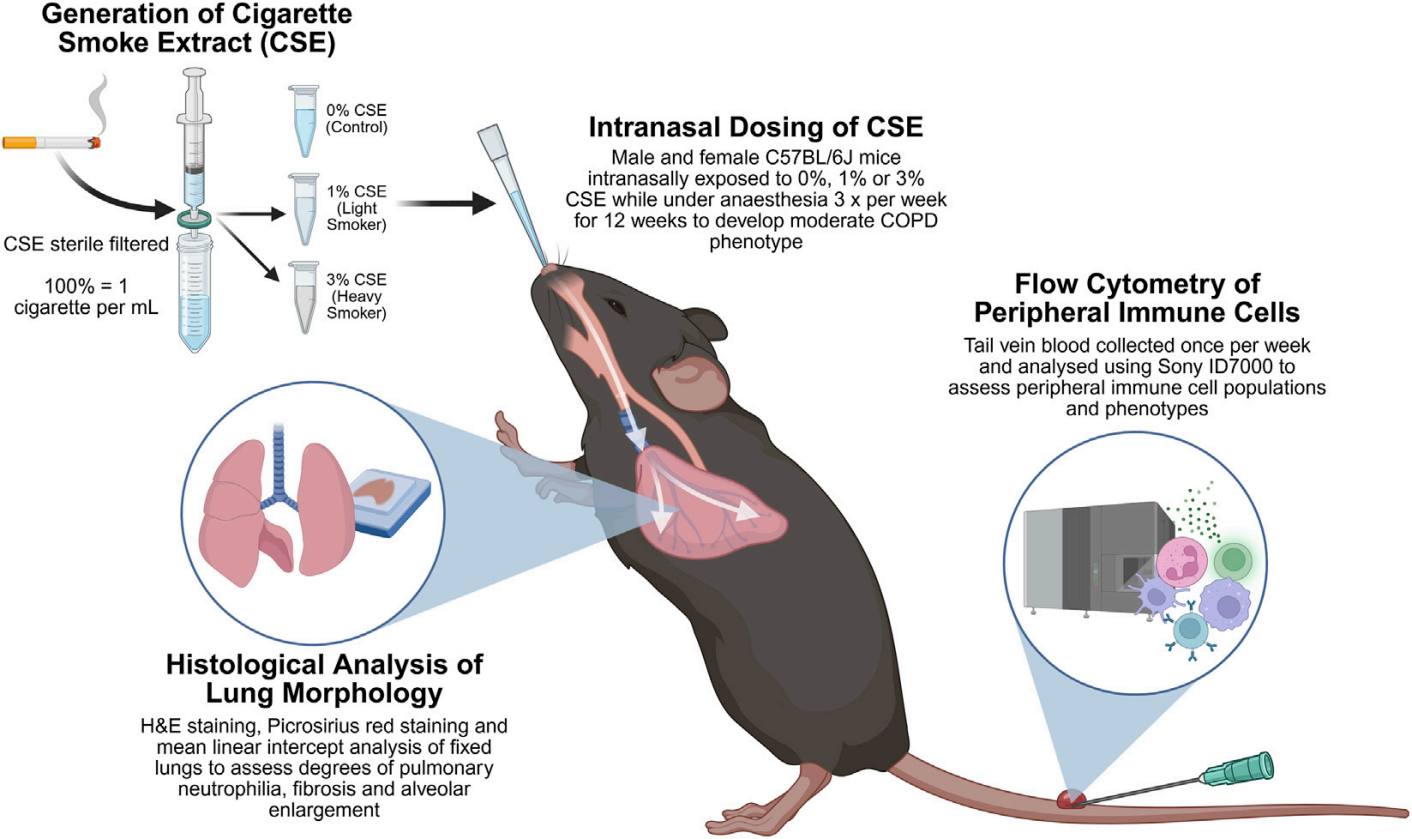
Diagrammatic overview of the study methodology. A 10% aqueous solution of CSE was generated using one cigarette bubbled into 10 mL phenol red-free RPMI, which was diluted to 1% (light smoker) and 3% (heavy smoker) CSE. A control vehicle comprising only of phenol red-free RMPI was given to control groups. Male and female C57BL/6J mice were anaesthetised and intranasally dosed with 25 μL CSE three times per week for 12 weeks. Throughout the exposure period, 20 μL blood was taken from the tail to assess peripheral immune cell populations phenotypes via spectral flow cytometry. At the end of the exposure period, mice were culled via intraperitoneal overdose and the lungs were excised and fixed for histological and immunohistochemical analysis to determine airway remodelling and immune cell infiltration.

An overview of the methodology used in the study is shown in Figure 1.

## Results

### Intranasal cigarette smoke exposure is well tolerated and does not induce sustained weight loss

No adverse clinical effects attributable to cigarette smoke exposure were observed throughout the 12-week intranasal exposure period. Reported welfare concerns were limited to behaviours consistent with normal murine activity, including overgrooming in females and transient fighting related to social hierarchy establishment in males.

**FIGURE 2:**
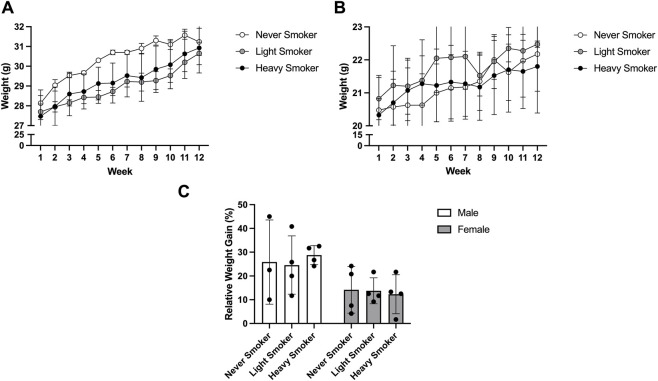
Average weight of male **(A)** and female **(B)** C57BL/6J mice per week during a 12-week exposure to CSE, and relative weight gain **(C)** of mice. Data shown as mean ± SEM (n = 3–4).

Body weight was monitored longitudinally as an indicator of systemic health. Both male and female mice across all exposure groups demonstrated consistent weight gain over the 12-week period (Figures 2A,B). No statistical significances were observed in relative weight gain across dose groups or sexes (Figure 2C). Collectively, these data indicate that intranasal cigarette smoke exposure was well tolerated and did not induce sustained weight loss or overt systemic distress, supporting its suitability for downstream analysis of immune and pathological responses.

### Intranasal cigarette smoke exposure induces selective alterations in peripheral immune populations

**FIGURE 3:**
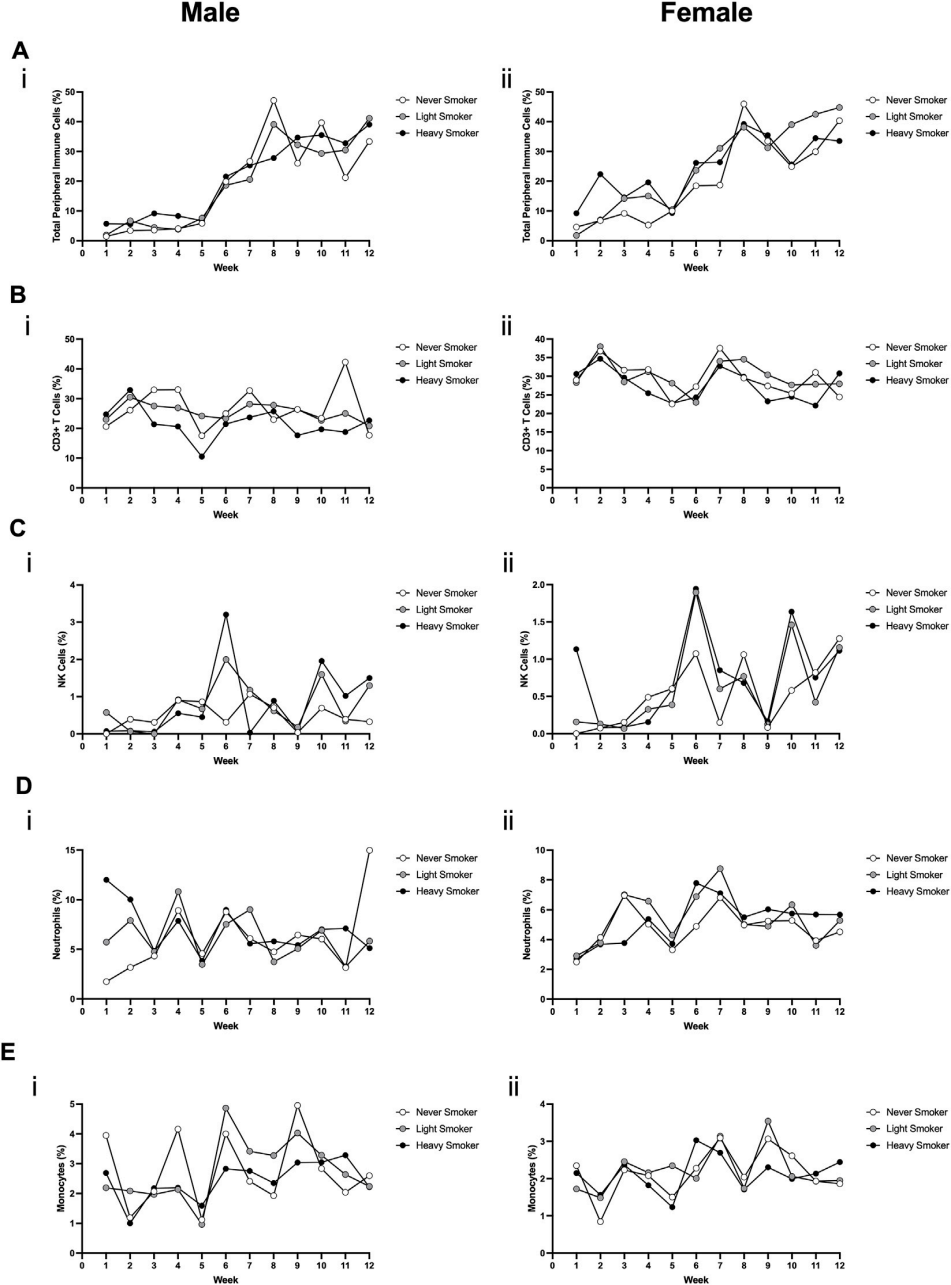
Time-course of total peripheral immune cells and different immune cell populations in male **(i)** and female **(ii)** C57BL/6J mice exposed to CSE. **(A)** Total Immune cells, **(B)** CD3^+^ T cells, **(C)** NK cells, **(D)** Neutrophils and **(E)** Monocytes. 0% CSE (Never Smoker; white dots), 1% CSE (Light Smoker; grey dots) and 3% CSE (Heavy Smoker; black dots). n = 3–4.

Peripheral immune cell populations were assessed longitudinally over the 12-week exposure period. Total peripheral immune cell frequencies exhibited modest temporal fluctuations in both male and female mice. However, these changes were not dose-dependent and were similarly observed in control animals, indicating that intranasal cigarette smoke exposure does not induce sustained global alterations in peripheral immunity (Figures 3Ai,ii).

Peripheral CD3^+^ T-cell frequencies displayed comparable temporal patterns across exposure groups, with no consistent dose-dependent effects observed in either sex (Figures 3Bi,ii). In contrast, selective alterations were observed within innate immune compartments. Peripheral natural killer (NK) cell frequencies increased over time, with more pronounced elevations in light and heavy smoker groups at later time points (Figures 3Ci,ii). Additionally, a modest increase in peripheral neutrophil frequencies was observed in heavy smoker mice during the late exposure period (weeks 8–12) (Figures 3Di,ii). Peripheral monocyte frequencies fluctuated over time but did not differ consistently between exposure groups (Figures 3Ei,ii).

Collectively, these data indicate that intranasal cigarette smoke exposure does not broadly disrupt peripheral immune homeostasis but is associated with selective changes in specific innate immune populations.

### Peripheral CD4^+^ T cells exhibit temporal activation-associated shifts independent of cigarette smoke dose

**FIGURE 4:**
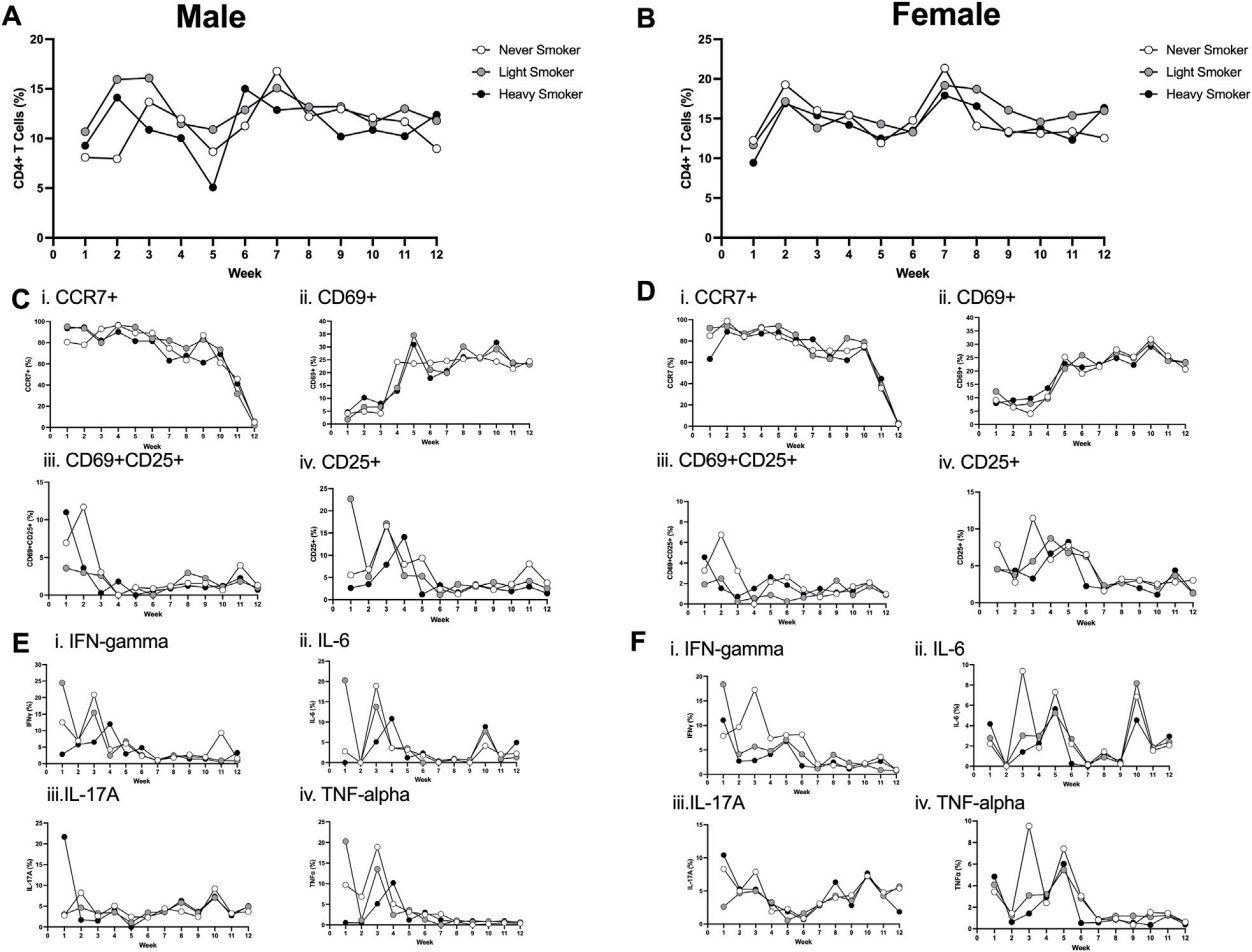
Time-course alterations to peripheral CD4^+^ T cells in male and female C57BL/6J mice exposed to CSE. Changes to total numbers peripheral CD4^+^ T cells over 12 weeks exposure period as a percentage of total peripheral immune cells **(A,B)** in mice exposed to 0% CSE (Never Smoker; white dots), 1% CSE (Light Smoker; grey dots) and 3% CSE (Heavy Smoker; black dots). Isolated populations were further phenotyped to examine percentage of CCR7+ **(Ci,Di)**, CD69^+^ **(Cii,Dii)**, CD69^+^CD25^+^ **(Ciii,Diii)** and CD25^+^ **(Civ,Div)** CD4^+^ T cells, with CD25^+^ cells classified as Tregs. Total numbers of cytokine positive CD4^+^ T cells were also determined for IFNy **(Ei,Fi)**, IL-6 **(Eii,Fii)**, IL-17A **(Eiii,Fiii)** and TNFa **(Eiv,Fiv)**. IL-17A+ CD4^+^ T cells were classified as Th17 cells. n = 3–4.

Phenotypic analysis of peripheral CD4^+^ T cells revealed temporal alterations in activation-associated markers over the 12-week exposure period in both male and female mice (Figure 4). Specifically, the percentage of CD4^+^ T cells stayed consistent in both male (Figure 4A) and female (Figure 4B) mice. Furthermore, in both male and female mice, the frequency of CCR7^+^ CD4^+^ T cells decreased progressively over time, while CD69^+^ CD4^+^ T-cell frequencies increased, consistent with a shift away from naïve phenotypes toward early activation states (Figures 4i,ii,i,ii). CD69^+^CD25^+^ CD4^+^ T cells were most abundant during the early exposure period before stabilising at lower levels, while CD25^+^CD69^-^ CD4^+^ T cells similarly declined following the initial weeks of exposure (Figures 4Eiii,iv,Fiii,iv). These trends were observed across all exposure groups, with no consistent dose-dependent effects.

Assessment of cytokine-expressing CD4^+^ T cells demonstrated transient increases during the early stages of the exposure period, with IFN-γ^+^, IL-6^+^, IL-17A^+^, and TNF-α^+^ CD4^+^ T-cell populations most prominent within the first half of the model before declining at later time points (Figures 4D,E). These temporal patterns were comparable across sexes and exposure groups, indicating that intranasal cigarette smoke exposure does not induce sustained, dose-dependent alterations in peripheral CD4^+^ T-cell effector function.

### Unbiased clustering analysis resolves major peripheral immune populations without sex-dependent differences

**FIGURE 5:**
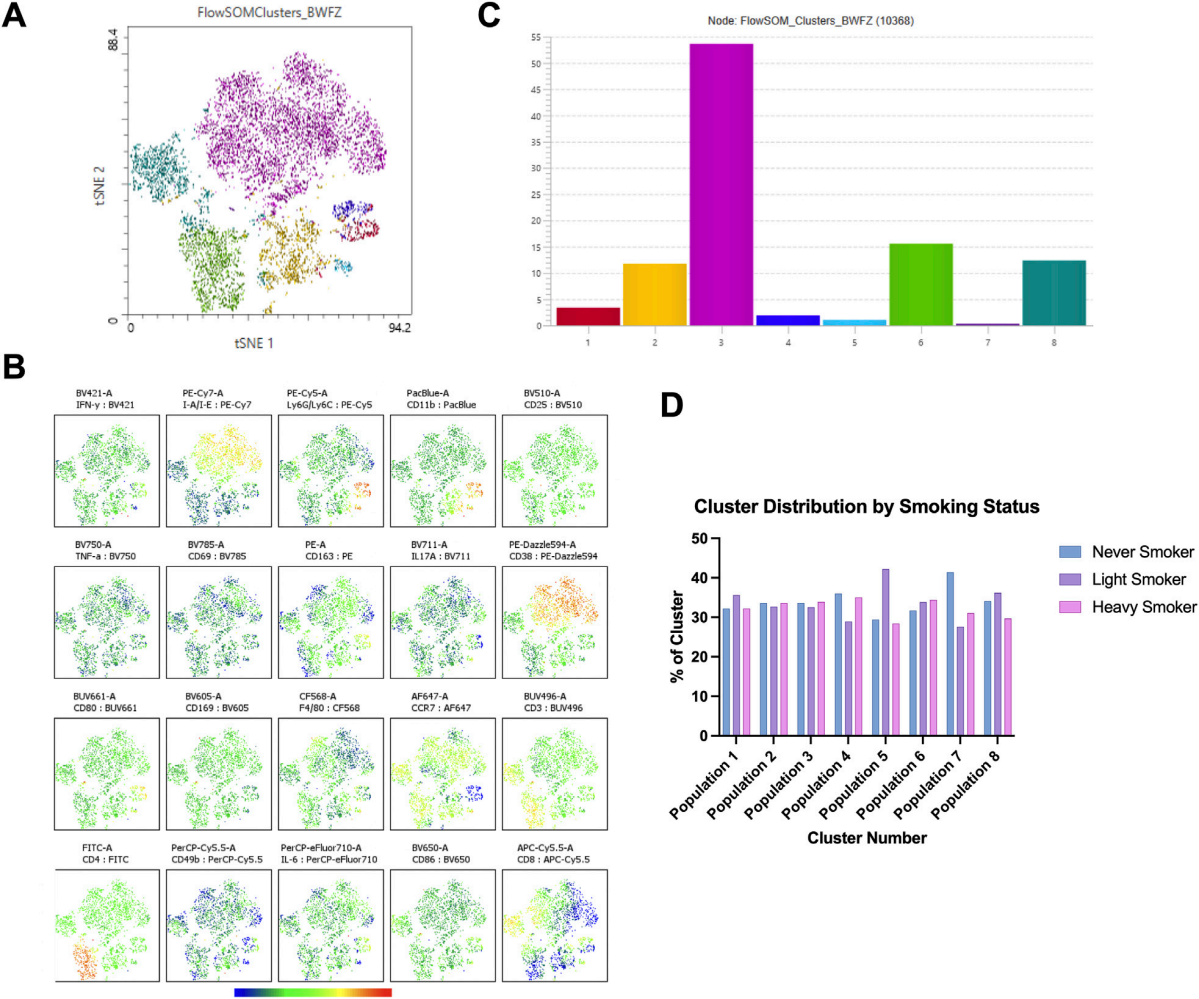
Unbiased clustering analysis of flow cytometry data. Data is presented as a tSNE plot **(A)**, as heatmap tSNE plots to identify location of each marker’s expression within clusters **(B)**, and the number of events in each FlowSOM cluster **(C)**. Data was then distinguished according to the percentage distribution of clusters according to disease group **(D)**. No statistical significances detected by way of 2way ANOVA analysis with p = < 0.05; n = 3.

To complement conventional gating strategies, an unbiased clustering approach was applied to peripheral CD45^+^ immune cells using FlowSOM and dimensionality reduction analysis (Figure 5). Figure 5A presents the tSNE visualization of FlowSOM clusters, Figure 5B is the tSNE visualization of marker density, and Figure 5C presents the percentage of CD45^+^ Immune cells found in each identified FlowSOM cluster, with cluster 3 comprising the largest proportion of events. Of the 8 clusters identified, there were multiple immune cell clusters corresponding to major lymphoid and myeloid populations, including CD4^+^ and CD8^+^ T cells, natural killer cells, and myeloid subsets, consistent with results obtained using manual gating strategies.

Assessment of cluster distribution across study variables revealed no significant differences in the relative abundance of immune cell populations between control, light smoker and heavy smoker groups. Although trends were observed for specific clusters, these did not reach statistical significance (Figure 5D). Together, these data demonstrate that unbiased clustering robustly captures expected peripheral immune cell populations and that CSE does not significantly influence peripheral immune cell composition in this model.

### Intranasal cigarette smoke exposure induces dose-dependent pulmonary neutrophilia and airway remodelling

**FIGURE 6:**
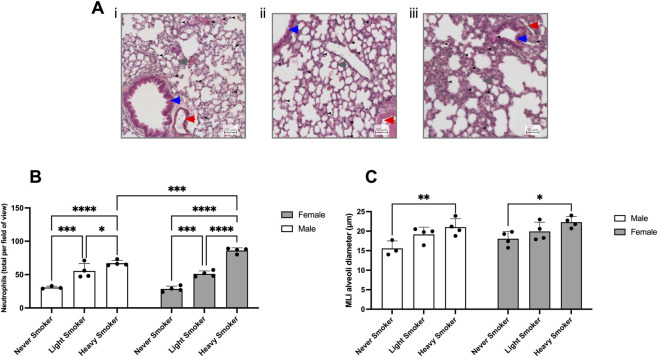
Airway remodelling and pulmonary neutrophilia in CSE exposed mice. **(A)** H&E staining of left middle lung sections containing alveoli, major airways and major or minor blood vessels of male C57BL/6J mice following 12 weeks of **(i)** 0%, **(ii)** 1% and **(iii)** 3% CSE exposure. Neutrophils are identified by black arrowheads, non-alveolar airways by blue arrowheads and blood vessels by red arrowheads. Scale bar = 50 μm; n = 3–4. **(B)** Total neutrophil counts in left middle lung sections of C57BL/6J mice following 12 weeks of CSE exposure. Total neutrophils were counted across a 500 × 500 μm section from the left middle lung lobe as an indication of pulmonary neutrophilia. **(C)** Mean linear intercept (MLI) analysis of C57BL/6J mice following 12 weeks of CSE exposure. Five random 500 × 500 μm sections from the left lung lobe were taken **(Ai)** and chord lengths of alveoli were measured horizontally **(Aii)** and vertically **(Aiii)** using the MLI plugin for ImageJ. Average alveolus diameter was then calculated from the resulting data. Statistics were performed using a 2way ANOVA. *p = <0.05; ***p = <0.001; ****p = <0.0001; n = 3–4.

Pulmonary neutrophil infiltration and structural lung pathology were assessed in histological sections of the left lung lobe (Figure 6). Compared to never-smoker controls, both light and heavy smoker mice exhibited increased neutrophil accumulation within airway regions, coinciding with evidence of airway remodelling (Figure 6A). Neutrophils were frequently localised to areas of apparent tissue damage and remodelling in cigarette smoke–exposed animals, with these features most pronounced in the heavy smoker groups.

Quantitative analysis demonstrated a significant, dose-dependent increase in pulmonary neutrophil counts following cigarette smoke exposure in both male and female mice (Figure 6B). In males, exposure to 1% and 3% CSE resulted in a significant increase in neutrophil numbers relative to controls, with a further significant increase observed between 1% and 3% CSE. Female mice exhibited a comparable significant increase at 1% and 3% CSE compared to controls, and again, a further significant increase was observed between 1% and 3% CSE. Interestingly, female heavy smokers (3% CSE) showed a significant increase in neutrophil infiltration compared to male heavy smokers (3% CSE).

Structural changes were further assessed by mean linear intercept (MLI) analysis as an indicator of emphysematous change. Chronic CSE exposure resulted in a dose-dependent increase in average alveolar diameter in both male and female mice, with significant enlargement observed in heavy smoker groups relative to never-smoker controls in both male and female groups (Figure 6C). These findings indicate that intranasal cigarette smoke exposure induces emphysematous lung remodelling in both sexes, accompanied by pronounced neutrophilic inflammation, particularly at higher exposure doses.

### Cigarette smoke exposure promotes collagen deposition and fibrotic airway remodelling

**FIGURE 7:**
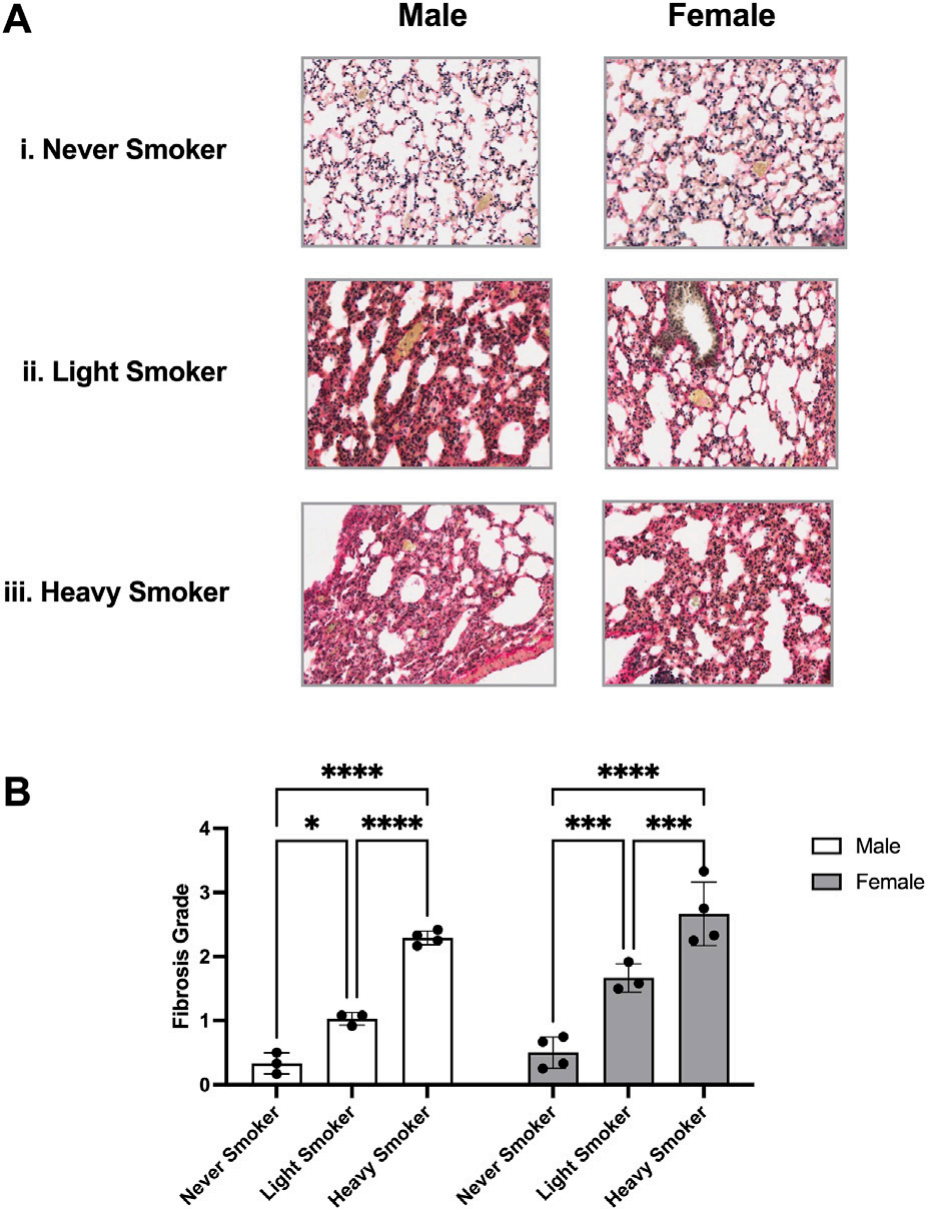
Picrosirius red staining of left middle lung sections from male and female C57BL/6J mice. **(A)** Representative histological images of murine lung tissue fibrosis across smoking exposure groups using a modified fibrosis grading scale (×10 magnification). **(B)** Murine lung tissue sections were assessed for fibrotic remodelling using a modified semi-quantitative fibrosis scale.

Collagen deposition within the lung was assessed by Picrosirius red staining of histological sections. Lung tissue sections were scanned using the Hamamatsu NanoZoomer and analysed using NDP.view2 software at ×10 magnification. Compared to never-smoker controls, cigarette smoke–exposed mice exhibited increased collagen deposition in a dose-dependent manner, predominantly localised to airway walls and perivascular regions (Figure 7A). These changes were most pronounced in heavy smoker animals, and coincided with regions exhibiting structural airway remodelling.

Pulmonary fibrosis was assessed using a modified semi-quantitative histopathological grading scale adapted from a previously established bleomycin-induced fibrosis model. This scale ranges from Grade 0 to Grade 8 according to the severity and extent of fibrotic lung remodelling. Grade 0 represents normal lung architecture with no detectable fibrosis. Grade 1 indicates isolated alveolar septa with mild fibrotic changes, while Grade 2 is characterised by fibrotic thickening of the alveolar septa with knot-like formation. Grade 3 reflects contiguous fibrotic walls of the alveolar septa. More advanced fibrosis is represented by Grade 4, showing single fibrotic masses, and Grade 5, showing confluent fibrotic masses. Grade 6 indicates large contiguous fibrotic masses, Grade 7 is characterised by air bubbles within fibrotic tissue, and Grade 8 represents severe fibrous obliteration of the lung structure. This modified grading system was used to compare the severity of fibrosis across all experimental groups.

Twenty-two histological images were independently evaluated by two observers. For each of the 22 images, five random fields were selected for assessment. Both observers had extensive experience in the histological evaluation of pulmonary fibrosis. This histopathological assessment of lung tissue demonstrated that fibrosis severity varied according to smoking exposure and sex. Both male and female mice in the heavy smoker groups exhibited the greatest fibrotic changes overall, showing a significantly higher fibrosis grade than both the never-smoker and light smoker groups (Figure 7B). In the low-smoking groups, fibrosis scores were reduced relative to high-smoking animals, with low-smoking females (LSF) demonstrating mild to moderate lesions (approximately Grade 1–2), while low-smoking males (LSM) showed milder changes closer to Grade 1, indicating isolated alveolar septa with gentle fibrotic alterations. Never-smoker control groups displayed the lowest fibrosis grades overall, with both never-smoker males and never-smoker females remaining near Grade 0–1, consistent with normal lung architecture or only minimal fibrotic change. Collectively, these findings suggest that smoking exposure increases pulmonary fibrosis in a dose-dependent manner, with female mice showing greater susceptibility than males under comparable exposure conditions.

## Discussion

Chronic cigarette smoke exposure is the primary aetiological driver of COPD, yet examining its immunopathogenesis in mice remains challenging due to welfare concerns and confounding systemic effects associated with traditional exposure paradigms. Using a refined intranasal CSE exposure method designed to minimise physiological stress, repeated CSE administration elicited clear, dose-dependent pathological changes in the lung, as demonstrated by increased pulmonary neutrophilia, airway remodelling, and collagen deposition, consistent with an emphysematous phenotype. These pathological features were most pronounced at higher exposure doses and were accompanied by sex-dependent differences in disease severity, with female mice exhibiting greater pulmonary inflammatory and structural pathology. In contrast, longitudinal immune profiling of peripheral blood revealed no consistent dose-dependent alterations in circulating immune cell populations or phenotypes, indicating that COPD-like lung pathology can develop in the absence of overt systemic immune disruption.

The dissociation between robust pulmonary pathology and stable peripheral immune profiles suggests that within this model, disease-relevant inflammatory processes are predominantly localised to the lung microenvironment. Pulmonary neutrophilia was the central feature of CSE-induced pathology observed, increasing with exposure dose and localising preferentially to regions of airway remodelling and apparent tissue damage. Neutrophil accumulation coincided with increased collagen deposition and alveolar enlargement, supporting a close spatial and temporal relationship between inflammation and structural lung injury. These findings are consistent with those of other studies, whereby neutrophils drive extracellular matrix degradation and loss of alveolar integrity through the release of proteases and inflammatory mediators (Benjamin et al., 2020; Wang et al., 2023). Importantly, these pathological features developed in the absence of systemic immune activation, reinforcing the interpretation that local inflammatory circuits within the lung are sufficient to promote COPD-like remodelling under repeated CSE exposure.

Despite extensive longitudinal sampling and the use of both conventional gating strategies and unbiased clustering analysis, peripheral immune cell populations remained broadly preserved across exposure groups. Temporal fluctuations occurred independently of CSE dose, likely reflecting age-associated immune variation rather than disease-specific effects. The immune system continually develops and later decays with age, a process known as inflammaging, which is widely reported as a contributing factor to many diseases of old age, including COPD (Zuo et al., 2019). In a comparison of aged (12-month-old) vs. young (2-month-old) mice chronically exposed to cigarette smoke for 12 weeks, aged mice displayed markedly increased pulmonary inflammation and airway remodelling in comparison to both control and young mice, implicating inflammaging as a significant risk factor in the development of COPD (John-Schuster et al., 2016). Although our findings did not demonstrate any changes in immune cell profiles across the 12-week exposure period, the mice were younger (11 weeks) at the start of the study and therefore had more robust immune systems than older mice. In the previous study by John-Schuster et al. (2016), systemic immune profiles were neither measured nor tracked, unlike in our study, which presents a possible avenue for further investigation, especially given the differences in lung immune profiles between aged and young mice.

To contextualise the contribution of our study, it is imperative to consider how previous murine models have approached immune characterisation in the context of cigarette smoke exposure. Our observation of no alterations to peripheral immune profiles contrasts with reports of systemic alterations in other murine cigarette smoke models, which demonstrate marked changes in immune cell distribution, differentiation and activation. This includes macrophage polarisation, enhanced plasmacytoid dendritic cell activation, and altered circulating T cell profiles (John-Schuster et al., 2016; De Cunto et al., 2020; Qiu et al., 2018). However, it is frequently reported in mouse models of COPD that exposure to, and effects generated by, cigarette smoke are not confined to the respiratory route. Mice in model systems employing whole-body or nose-or-head only exposure methods are routinely exposed to particulate matter from cigarette smoke via the skin, which may be ingested through grooming or directly absorbed by the skin itself, which may artificially amplify or distort systemic immune signals (Serre et al., 2021; Nemmar et al., 2012). Furthermore, chronic restraint processes required for such model systems are reported to increase markers of stress, leading to weight loss and systemic immune changes, which may impact the findings of previous murine models of COPD (Shoji and Miyakawa, 2020; Zhu et al., 2019). By eliminating the need for prolonged restraint and ensuring direct exposure of CSE in a biologically relevant manner applicable to human disease, the intranasal approach used in this study bypasses many of these confounding factors. Prior intranasal CSE models have demonstrated pulmonary inflammatory changes, including BAL neutrophilia, macrophage recruitment, and airway cytokine elevations, but were characterised over relatively short exposure periods and did not include longitudinal peripheral immune profiling (Lamb et al., 2012; Miller et al., 2002). Furthermore, some models utilising an intranasal dosing strategy induce COPD via the use of lipopolysaccharide or porcine pancreatic elastase (PPE), which may not always encapsulate a COPD phenotype observed in humans (Shen et al., 2025). Our study addresses this gap by combining a refined, stress-minimised intranasal delivery approach with concurrent longitudinal characterisation of both pulmonary pathology and systemic immune profiling. In doing so, we demonstrate that COPD-like pulmonary pathology can develop and progress in the absence of detectable systemic immune dysregulation, a finding with implications for how the compartmentalisation of inflammatory responses in early or moderate COPD is understood.

Several limitations in our study should nevertheless be acknowledged. Group sizes were necessarily small due to ethical constraints during model optimisation, and repeated peripheral blood sampling volumes limited the depth of systemic immune analyses. Additionally, immune profiling was restricted to circulating cells, and future studies incorporating lung-resident immune populations through bronchoalveolar lavage fluid (BALF) analysis would provide valuable mechanistic insight. Despite these limitations, the model consistently generated dose-dependent pulmonary pathology and revealed sex-specific differences in disease severity. As such, intranasal CSE exposure represents an amenable and refined platform for investigating pulmonary immunopathology and for future studies examining disease modifiers, sex-dependent mechanisms, and therapeutic interventions.

## Data Availability

The original contributions presented in the study are included in the article/Supplementary Material, further inquiries can be directed to the corresponding author.
