# Supplementary material for: Impact of intranasal cigarette smoke exposure on murine peripheral immune responses and lung pathology
Source: Front Toxicol. 2026 Aug 3;8:1884554. doi: 10.3389/ftox.2026.1884554 (PMC13477786; doi:10.3389/ftox.2026.1884554)
Supplement: Supplementary file 1 [file Supplementaryfile1.docx]

Supplementary File 1

Calculations were made to determine the percentage of CSE to be used. This was performed by calculating the number of cigarettes smoked per day per gram of average human weight, and calculating how many cigarettes smoked per week per average weight of an 8-week-old mouse in percentage form. While this method does not account for factors such as metabolic and lung structure differences, this method provides a justifiable reasoning behind the concentrations mice were exposed to. As such, the following calculations were performed to determine CSE doses:

$$no. cigarettes per day \div average human weight \left( kg \right) =cigarettes per kg$$

$$cigarettes per kg \div1000 =cigarettes per gram \left( g \right)$$

$$cigarettes per g \times average weight of 8 week old mouse \left( g \right)=no. cigarettes per mouse$$

$$\left( no. cigarettes per mouse \times7 \right)\times100 = \% of cigarette smoke extract required$$

$$Light Smoker Males:$$

$$5 \div84.5 =0.05855 cigarettes per kg$$

$$0.05855 \div1000 =0.00005855 cigarettes per g$$

$$0.00005855 \times25=0.001464$$

$$\left( 0.001464 \times7 \right)\times100 = 1.02 \%$$

$$Light Smoker Females:$$

$$5 \div72.1 =0.06935 cigarettes per kg$$

$$0.06935 \div1000 =0.00006935 cigarettes per g$$

$$0.00006395 \times20=0.001387$$

$$\left( 0.001387 \times7 \right)\times100 = 0.97 \%$$

$$Heavy Smoker Males:$$

$$15 \div84.5 =0.17564 cigarettes per kg$$

$$0.17564 \div1000 =0.00017564 cigarettes per g$$

$$0.00017564 \times25=0.004391$$

$$\left( 0.004391 \times7 \right)\times100 = 3.07 \%$$

$$Heavy Smoker Females:$$

$$15 \div72.1 =0.20804 cigarettes per kg$$

$$0.20804 \div1000 =0.00020804 cigarettes per g$$

$$0.00020804 \times20=0.0041608$$

$$\left( 0.0041608 \times7 \right)\times100 = 2.91 \%$$
