# Supplementary material for: Impact of intranasal cigarette smoke exposure on murine peripheral immune responses and lung pathology
Source: Front Toxicol. 2026 Aug 3;8:1884554. doi: 10.3389/ftox.2026.1884554 (PMC13477786; doi:10.3389/ftox.2026.1884554)
Supplement: Supplementary file 2 [file Supplementaryfile2.docx]

**Supplementary File 2**

**Gating strategy for identification of peripheral immune cell populations**

Multiparametric spectral flow cytometry was performed on whole blood using a 22-colour antibody panel. Following acquisition on the Sony ID7000, immune cell populations were identified using a hierarchical gating strategy applied uniformly across all samples (Supplementary Fig. 1).

Events were sequentially gated to exclude debris, doublets, and non-viable cells, followed by selection of CD45⁺ leukocytes (Supplementary Fig. 1A). CD45⁺ cells were then subdivided into CD3⁺ and CD3⁻ populations to enable lineage-specific downstream gating.

**T cell identification, activation, and cytokine expression**

T cells were defined as CD45⁺CD3⁺ cells and further subdivided into CD4⁺ and CD8⁺ subsets (Supplementary Fig. 1B.i – ii). Activation status was assessed using CCR7, CD69, and CD25 expression. CD4⁺ and CD8⁺ T cells were classified as CCR7⁺ (naïve/central memory), CD69⁺CD25⁻ (early activation), CD69⁺CD25⁺ (intermediate activation), or CD69⁻CD25⁺ (late activation) (Supplementary Fig. 1C.i – ii). Within the CD4⁺ compartment, CD69⁻CD25⁺ cells were considered to include regulatory T cell–enriched populations in addition to activated CD4⁺ T cells.

Cytokine-producing T cells were identified following intracellular staining, with IFN-γ, IL-6, TNF-α, and IL-17A assessed within both CD4⁺ and CD8⁺ subsets (Supplementary Fig. 1D). CD4⁺IL-17A⁺ cells were considered to represent Th17-enriched populations.

**NK cells, monocytes, and neutrophils**

Non-T cell populations were identified within the CD45⁺CD3⁻ compartment. NK cells were defined as CD49b⁺ cells and subsequently analysed for intracellular cytokine expression (Supplementary Fig. 1B.iii).

Neutrophils and monocytes were distinguished using CD11b and Ly6G/Ly6C (Gr-1). Neutrophils were defined as CD11b⁺Gr-1^hi cells, while monocytes were defined as CD11b⁺Gr-1^med cells (Supplementary Fig. 1B.iv). Both populations were further gated for cytokine expression.

**Macrophages, dendritic cells, and B cells**

Dendritic cells (DCs) and B cells were gated from CD45⁺CD3⁻MHCII⁺CD11b⁻ cells. Within this population, CCR7 and CD38 expression was used to distinguish DCs (CCR7⁺CD38⁺) from B cells (CCR7⁻CD38⁺) (Supplementary Fig. 1B.vi). Both populations were further assessed for cytokine expression.

Macrophages were identified within the CD45⁺CD3⁻ compartment by sequential gating on MHC class II (I-A/I-E) and CD11b expression. MHCII⁺CD11b⁺ cells were further gated for CD80 and CD86 co-expression, followed by selection of CD163⁺ cells, which were defined as macrophage populations (Supplementary Fig. 1B.vii – viii). Identified macrophages were subsequently analysed for intracellular cytokine expression.

Supplementary Figure 1:- Population definition and phenotyping of peripheral immune cells. Immune cells were gated according to size and granularity (A.i), single cells (A.ii), live (A.iii) and CD45+ (A.iv). Using this population, CD45+ immune cells were gated for CD3 (B.i), with CD3+ populations used to distinguish CD4+ and CD8+ T cell subsets (B.ii). The CD3- population was then used to identify NK cells (B.iii) and monocytes and neutrophils (B.iv). CD3- cells were also gated for I-A/I-E and CD11b (B.v) to determine DC and B cell populations (B.vi) as well as macrophages (B.vii - viii). T cells were phenotyped according to activation markers including CCR7 (C.i) and CD69/CD25 (C.ii), which were used to determine early/late stage of activation. CD4+CD69-CD25+ T cells were defined as Tregs. All cells were gated for cytokines IFNy (D.i), IL-6 (D.ii), IL-17A (D.iii) and TNFa (D.iv). CD4+IL-17A+ T cells were defined as Th17 cells.
