# Supplementary material for: Impact of intranasal cigarette smoke exposure on murine peripheral immune responses and lung pathology
Source: Front Toxicol. 2026 Aug 3;8:1884554. doi: 10.3389/ftox.2026.1884554 (PMC13477786; doi:10.3389/ftox.2026.1884554)
Supplement: Supplementary file 3 [file Table1.docx]

Supplementary Table 1 - **22-Colour Flow Cytometry panel.** Details of all antibodies, including conjugate, brightness (according to supplier’s specifications), company purchased from, catalogue number and concentration used during analysis. Purified antibodies were conjugated in-house using fluorophore conjugation kits as detailed in Supplementary Table 2.

| Antibody | Fluorophore | Brightness | Clone | Company | Cat No. |
| --- | --- | --- | --- | --- | --- |
| **CD45** | APC-Fire810 | 1 | 30-F11 | Sony Biotechnology | 1115865 |
| **CD3** | BUV496 | 2 | 17A2 | BD Biosciences | 741117 |
| **CD4** | FITC | 3 | RM4-5 | Sony Biotechnology | 1102550 |
| **CD8a** | APC/Cy5.5 | 3 | 53-6.7 | Abcam | ab82005 |
| **CD25** | BV510 | 3 | PC61 | Biolegend | 102042 |
| **CD49b** | PerCP/Cy5.5 | 3 | DX5 | Biolegend | 108915 |
| **CD11b** | Pacific Blue | 1 | M1/70 | Sony Biotechnology | 1106120 |
| **Ly6G/Ly6C** | PE/Cy5 | 5 | RB6-8C5 | Sony Biotechnology | 1142050 |
| **I-A/I-E** | PE/Cy7 | 4 | M5/114.14.2 | Sony Biotechnology | 1138150 |
| **F4/80** | Purified | - | T45-2342 | BD Biosciences | 565409 |
| **CD163** | PE | 5 | S15049F | Sony Biotechnology | 1383515 |
| **CD169** | BV605 | 4 | 3D6.112 | Biolegend | 142413 |
| **CD80** | BUV661 | 3 | 16-10A1 | BD Biosciences | 741515 |
| **CD86** | BV650 | 3 | GL-1 | Sony Biotechnology | 1125175 |
| **CD38** | PE/Dazzle 594 | 5 | 90 | Sony Biotechnology | 1113650 |
| **CD69** | BV785 | 3 | H1.2F3 | Sony Biotechnology | 1122715 |
| **CCR7** | AF647 | 4 | 4B12 | Sony Biotechnology | 1200545 |
| **IFN-γ** | BV421 | 5 | XMG1.2 | Sony Biotechnology | 3129150 |
| **IL-6** | PerCP-efluor 710 | 4 | MP5-20F3 | Thermo Fisher | 46-7061-82 |
| **IL-17A** | BV711 | 3 | TC11-18H10.1 | Sony Biotechnology | 3134705 |
| **TNF-α** | BV750 | 3 | MP6-XT22 | Biolegend | 506358 |
| **Zombie** | NIR | 4 | - | Biolegend | 423106 |
