# Supplementary material for: Impact of intranasal cigarette smoke exposure on murine peripheral immune responses and lung pathology
Source: Front Toxicol. 2026 Aug 3;8:1884554. doi: 10.3389/ftox.2026.1884554 (PMC13477786; doi:10.3389/ftox.2026.1884554)
Supplement: Supplementary file 4 [file Table2.docx]

Supplementary Table 2 - **Conjugation kit details for in-house labelled antibodies.** Dye:antibody ratio was confirmed via NanoDrop spectrophotometry unless extinction coefficients could not be obtained due to restrictions as a result of proprietary information.

| Marker | Fluorophore | Brightness | Company | Cat No. | Dye:Antibody Ratio |
| --- | --- | --- | --- | --- | --- |
| **F4/80** | CF568 | 4 | Sigma-Aldrich | MX568S100-1KT | 3:1 |
